# Endodontic Microbiology: A Bibliometric Analysis of the Top 50 Classics

**DOI:** 10.1155/2021/6657167

**Published:** 2021-06-01

**Authors:** Mohmed Isaqali Karobari, Manahil Maqbool, Paras Ahmad, Muqthadir Siddiqui Mohammed Abdul, Anand Marya, Adith Venugopal, Gul Muhammad Shaik, Giuseppe Alessandro Scardina, Pietro Messina, Tahir Yusuf Noorani

**Affiliations:** ^1^Conservative Dentistry Unit, School of Dental Sciences, Universiti Sains Malaysia, Health Campus, 16150 Kubang Kerian, Kota Bharu, Kelantan, Malaysia; ^2^Paediatric Dentistry Unit, School of Dental Sciences, Universiti Sains Malaysia, Health Campus, 16150 Kubang Kerian, Kota Bharu, Kelantan, Malaysia; ^3^Department of Oral Medicine, Dental section, Akhtar Saeed Medical and dental college, Lahore 53720, Pakistan; ^4^Pediatric Dentistry, Department of Dental Services, King Khaled Hospital and Prince Sultan Centre for Health Sciences, Al Kharj, Riyadh, Saudi Arabia; ^5^Department of Orthodontics, Faculty of Dentistry, University of Puthisastra, Phnom Penh, Cambodia; ^6^Department of Orthodontics, Saveetha Dental College, Saveetha Institute of Medical and Technical Sciences, Chennai, India; ^7^Department of Dental Education and Research, Shahida Islam Medical and Dental College Lodhran, Pakistan; ^8^Department of Surgical, Oncological and Stomatological Disciplines, University of Palermo, Italy

## Abstract

**Background:**

Citation analysis has emerged to play a significant role in recognition of the most useful areas of research. Endodontic microbiology has been a topic of interest for endodontists as well as periodontists and oral surgeons. This bibliometric analysis is aimed at identifying and reporting the characteristics of the top 50 cited articles on endodontic microbiology.

**Methods:**

The articles were identified through a search on Web of Science (WoS), property of Clarivate Analytics database published on endodontic microbiology. The citation information of the selected articles was recorded. The *Journal of Endodontics*, *International Endodontic Journal*, *Oral Surgery Oral Medicine Oral Pathology Oral Radiology and Endodontology*, *Dental Traumatology*, and *Australian Endodontic Journal* were searched in the search title. Descriptive and bivariate analyses were performed using a statistical software package SPSS. Statistical analysis was performed using Shapiro-Wilk, Kruskal-Wallis, Post hoc, Mann-Kendall trend, and Spearman-rank tests.

**Results:**

The 50 most cited articles were published from 1965 to 2012 with citation count varying from 1065 to 103 times. The total citation counts of articles recorded were 11,525 (WoS), 12,602 (Elseviers' Scopus), and 28,871 (Google Scholar). The most prolific years in terms of publications were 2001, 2002, and 2003, with five publications each, followed by 2005 with four. The year with most citations was 1998, with 1,330 citations, followed by 1965 and 2001, with 1,065 and 1,015 citations, respectively. A total of 136 authors contributed to the top 50 most cited articles with 27 corresponding institutions from 12 different countries. The most common methodological design was *in vitro* study, followed by clinic-laboratory study, literature review, systematic review and meta-analysis, and animal study.

**Conclusions:**

The present study provided a detailed list of the top 50 most cited and classic articles on microbiology in endodontics. This will help researchers, students, and clinicians in the field of endodontics as an impressive source of information.

## 1. Introduction

Endodontic microbiology is a vast, interesting, and widely explored area of dentistry [1–4]. The pulpal and peri-radicular infections are mediated by the formation of biofilm [5]. This biofilm is located in areas that are inaccessible to mechanical cleansing of the root canal system. If these pathogenic microbes persist and are not appropriately removed [2, 4, 5], there is a high chance of postendodontic treatment failure [6, 7]. It has been suggested that every endodontic infection is polymicrobial, i.e., it is a collective outcome of multiple microbes which leads to pathogenesis [8]. Hence, understanding the nature of this polymicrobial infection is very important to ensure its removal and success of endodontic treatment.

In this advancing era of digital libraries and the availability of diverse research materials in the fields of dentistry from different parts of the world, citation analysis has emerged to play a significant role in recognition of the most useful areas of research. Endodontic microbiology has been a topic of interest not only for endodontists but also for periodontists and oral surgeons. It is a field that requires a multidisciplinary approach, involving contributions from different parts of the world and by authors of different origins and institutions. Hence, a bibliometric analysis makes it easier for students, budding researchers, or academicians to identify the articles that are most commendable in their field of interest [9]. This analysis not only provides information on the most cited articles but also helps in retrieving highly impactful research in the related field [10]. The first article on endodontic microbiology first appeared in the month of September 1965 in PubMed, followed by in the year 1987, 1989, 1990 to 1997, and so on.

Classics have been defined differently by multiple authors. The founder of the institution of Scientific Information (ISI), Dr. Eugene Garfield, poised the term “citation classics.” The purpose of this was to help acknowledge the frequently cited and peer-reviewed research [11]. A study conducted by researchers evaluated the bibliometrics' predictive ability to calculate the citation rate [12]. A work was referred to as a classic in some fields if it was cited more than 100 times [13–15], while in others it was mandatory to receive 400 citations and above to achieve the title of being called a classic [3]. In the last decade, citation analysis has become a very common tool of interest in both the fields of medicine [16–18] and dentistry [3, 13, 19–24]. However, to the best of our knowledge, citation analysis of the top 50 most cited classic articles in endodontic microbiology has not been carried out. This article is aimed at identifying and analysing the top 50 most cited classic articles in the field of endodontic microbiology.

## 2. Methods

### 2.1. Search Methodology and Data Source

The Web of Science (WoS), property of Clarivate Analytics database, was utilized to retrieve the data on articles published on microbiology in endodontics and their citation information. The data search was performed using (https://www.webofknowledge.com) all database on 1st of July 2020; the top journals in endodontics, *Journal of Endodontics*; *International Endodontic Journal*; *Oral Surgery, Oral Medicine, Oral Pathology, Oral Radiology, and Endodontology*; *Dental Traumatology*; and *Australian Endodontic Journal*, were searched in the search title. There was no restriction in the search regarding the publication year and the design of the study.

The top 50 most cited articles were selected and were arranged according to the decreasing number of their citation count. Manual reviewing of the selected articles was performed by the two authors independently by accessing the abstract and full-length if required; any disarrangement between them was solved with the consultation from the third author. The citation count of the top 50 articles was searched and recorded using Elsevier's Scopus (ES) and Google Scholar (GS). The current density of the articles was also calculated by dividing the total number of citations achieved with the number of the years since publication.

### 2.2. Data Extraction

The article title, article citation count, publication year, study design, financial support, name of first author and coauthors, country of origin, corresponding institution, and the keywords of the top 50 most cited articles were recorded. The country of origin and the institute of articles were determined by the address published as correspondence and reprints.

### 2.3. Journal Metrics

Three indicators, i.e., 5-year journal impact factor (http://www.jcr.clarivate.com), CiteScore (http://www.journalmetrics.scopus.com), and Eigenfactor score (http://www.eigenfactor.org), were used to determine the relative position of journals.

1. *Five-Year Journal Impact Factor*. This indicator represents the citation counts received by a journal, in one year, of the citable papers published in the last 5 years. Its calculation follows the following formula: citations from journal citation report (JCR) year of documents published in the last 5 years divided by the total number of citable documents [25]
2. *CiteScore (CS)*. This is a newly introduced indicator adopted to assess the impact of journals so that more rigorous results can be obtained. Its calculation follows the following formula: the ratio of citation counts from all items in 1 year to all items published over the past 3 years for a journal [25, 26]
3. *Eigenfactor Score (ES)*. This is regarded as an indicator of the global repercussions or impact of documents published online in JCR. Its calculation is based on the citation counts of items published in the past 5 years in the JCR per annum. It also takes into account which journals have contributed to these citations, so that highly cited journals will impact the network greater than lesser cited journals; references from one paper to another paper from the same journal are eliminated, so that ES is not biased by journal self-citation [25, 26]

### 2.4. Data and Statistical Analysis

The Visualization of Similarities viewer (VOSviewer) software [27] was used to create collaboration network maps regarding the cooccurrences of all keywords.

Statistical analysis was performed using software package, i.e., IBM SPSS Statistics version 24.0 (IBM, Chicago, IL). Shapiro-Wilk test was used to assess the normality of the data; based on normality and distribution of data, the mean standard deviation was calculated. The Kruskal-Wallis test was performed to assess the median differences between the independent groups. Post hoc testing was conducted to evaluate the median differences within each group. The Mann-Kendall trend test was performed to investigate any increase or decrease in the time-dependent trends. The correlation between the age of the journal and the publication count of the journal were evaluated using the Spearman-rank test. The *p* < 0.05 value was considered statistically significant.

## 3. Results

### 3.1. Citation Count, Citation Density, and Current Citation Index

The primary characteristics of the top 50 most cited articles are shown in Table 1. The citation counts of the top 50 publications varied from 103 to 1065 (median, 171.0), with a total citation count of 11,525 (WoS), from 97 to 1202 (median, 187.5), with a total citation count of 12,602 (ES), and from 202 to 2762 (median, 461.5), with a total citation count of 28,871 (GS). The most cited article, with a total of 1065 (WoS), 1202 (ES), and 2762 (GS) citations, was titled “The Effects of Surgical Exposures of Dental Pulps in Germ-Free and Conventional Laboratory Rats” [28] and was published in the *Oral Surgery, Oral Medicine, Oral Pathology, Oral Radiology, and Endodontology*. Its citation density (CD) was 19.72, with the current citation index (CCI) of 41. The second most cited article, with a total of 791 (WoS), 839 (ES), and 1883 (GS) citations, was titled “Microbiologic Analysis of Teeth with Failed Endodontic Treatment and the Outcome of Conservative Re-treatment” [29] and also was published in the *Oral Surgery, Oral Medicine, Oral Pathology, Oral Radiology, and Endodontology*. Its CD was 37.67, with the CCI of 26. The third most cited article, with a total of 539 (WoS), 576 (ES), and 1269 (GS) citations, was titled “Microbiological Status of Root-Filled Teeth with Apical Periodontitis” [30] and was published in the *International Endodontic Journal*. Its CD was 25.67, with the CCI of 14. According to the CCI 2019, the top-ranked article was the literature review published in 2008, securing 321 citations [31]. As per CD, the clinic-laboratory study by Sundqvist et al. [29] has the highest score, i.e., 37.67.

**Figure 1:**
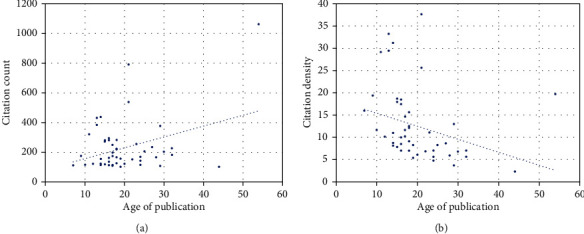
Association of (a) citation count and (b) citation density with age of publication.

The age of the article has shown a nonsignificant relation towards the higher citation count (*r* = 0.011, *p* = 0.840) (Figure 1(a)). Further, a significant negative trend towards an increased citation density was observed (*r* = −0.482, *p* < 0.05) (Figure 1(b)). According to the Shapiro-Wilk test, the distribution of data regarding citation count, citation density, and article age was not normal (*p* < 0.05).

### 3.2. Distribution by Year

**Figure 2:**
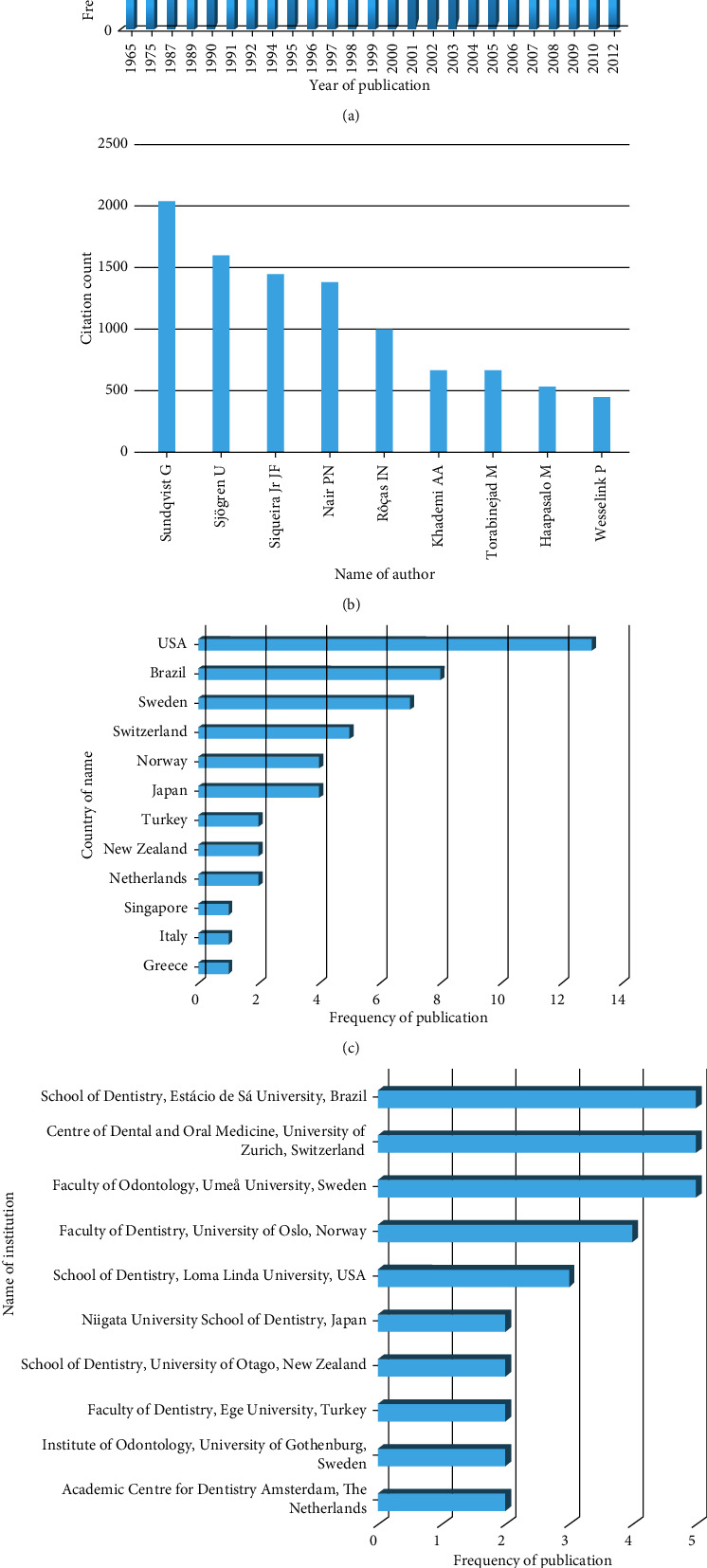
(a) Citation analysis of the top 50 most cited articles over the years. Contribution of (b) authors, (c) countries, and (d) institutions to the top 50 most cited articles.

The top 50 most cited articles were published between 1965 [28] and 2012 [32] (Figure 2(a)). The most prolific years in terms of publications were 2001, 2002, and 2003, with five publications each, followed by 2005 (*n* = 4). The year with most citations was 1998, with 1,330 citations, followed by 1965 and 2001, with 1,065 and 1,015 citations, differently. The decade with most publications (*n* = 29) and citations (*n* = 5,976) was 2000s.

### 3.3. Authors

The top 50 most cited articles in endodontic microbiology were contributed by 136 authors. The major contribution was made by Siqueira Jr JF (*n* = 7, 1441), followed by Sundqvist G (*n* = 6, 2039 citations), Sjögren U (*n* = 4, 1597), Nair PN (*n* = 4, 1379), Rôças IN (*n* = 4, 995), Khademi AA (*n* = 3, 665), Torabinejad M (*n* = 3, 665), Haapasalo M (*n* = 3, 527), and Wesselink P (*n* = 3, 447) (Figure 2(b)).

### 3.4. Countries and Institutions

Twelve countries contributed in the top 50 most cited articles in endodontic microbiology which includes Brazil, Greece, Italy, Japan, Netherlands, Norway, New Zealand, Singapore, Sweden, Switzerland, Turkey, and the United States of America (Figure 2(c)). Depending on the number of articles, most of the articles contributed from the United States of America (*n* = 13, 3208 citations), followed by Brazil (*n* = 8, 1740 citations), Sweden (*n* = 7, 2313 citations), Switzerland (*n* = 5, 1496 citations), Norway (*n* = 4, 641 citations), Japan (*n* = 4, 593 citations), New Zealand (*n* = 2, 457 citations), Turkey (*n* = 2, 311 citations), Netherlands (*n* = 2, 279 citations), Singapore (*n* = 1, 154 citations), Greece (*n* = 1, 118 citations), and Italy (*n* = 1, 112 citations).

According to the affiliation of the corresponding authors, a total of 27 institutions were affiliated. The major institutions, with five publications each, were the Faculty of Odontology, Umeå University, Sweden; Centre of Dental and Oral Medicine, University of Zurich, Switzerland; and School of Dentistry, Estácio de Sá University, Brazil, followed by Faculty of Dentistry, University of Oslo, Norway (*n* = 4) and School of Dentistry, Loma Linda University, USA (*n* = 3) (Figure 2(d)).

### 3.5. Journals

**Figure 3:**
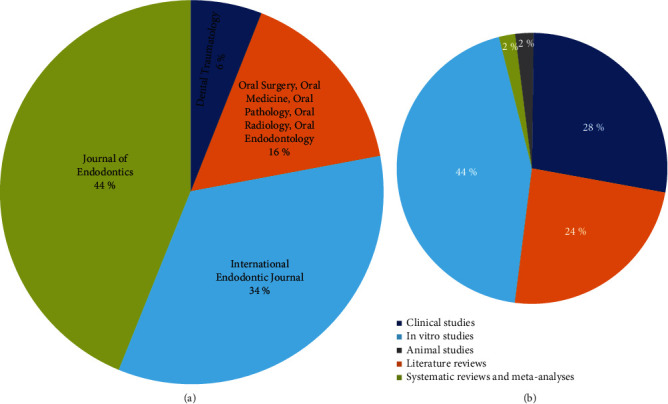
(a) Contribution of journals to the top 50 most cited articles. (b) Distribution of study design of the top 50 most cited articles.

The top 50 most cited articles in endodontic microbiology were published in four different journals. The *Journal of Endodontics* (*n* = 22) was with the most number of publications, followed by the *International Endodontic Journal* (*n* = 17), *Oral Surgery, Oral Medicine, Oral Pathology, Oral Radiology and Endodontology* (*n* = 8), and *Dental Traumatology* (*n* = 3) (Figure 3(a) and Table 2). *Journal of Endodontics* had the highest citation count (*n* = 4190), followed by the *International Endodontic Journal* (*n* = 3440), *Oral Surgery, Oral Medicine, Oral Pathology, Oral Radiology and Endodontology* (*n* = 3419), and *Dental Traumatology* (*n* = 476).

The number of “classic” articles published in that journal has shown a statistically significant trend (*p* < 0.01) in relation with the age of the journal. Though, a statistically nonsignificant trend (*p* = 0.348) was observed in relation with the impact factor of the journal. According to the simple linear regression analysis, a statistically significant association was observed between self-citation (*p* = 0.041), CiteScore (*p* = 0.033), Eigenfactor score (*p* = 0.006), and total citation count (Table 3).

### 3.6. Methodological Design

The most common methodological design in the top 50 publications was *in vitro* study (*n* = 22) (3905 citations), followed by clinic-laboratory study (*n* = 14) (3692 citations), literature review (*n* = 12) (2741 citations), systematic review and meta-analysis (*n* = 1) (122 citations), and animal study (*n* = 3) (1065 citations) (Figure 3(b)). No statistical significance was detected (*p* = 0.760) while analysing the median difference in the citation count per publication among *in vitro* study, clinic-laboratory study, literature review, systematic review and meta-analysis, and animal study.

### 3.7. Evidence Level

Out of the total five evidence levels (ELs) [33], the top 50 most cited publications could primarily be classified into three ELs. Most of the articles were within evidence level V (*n* = 35), followed by EL III (*n* = 14) and EL I (*n* = 1). Among these ELs, the total citation counts (*r* = −0.2310, *p* = 0.076) and the citation density (*r* = 0.122, *p* = 0.436) did not vary significantly.

### 3.8. Keywords

**Figure 4:**
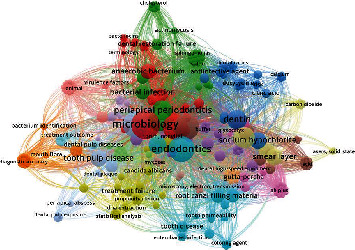
Keywords identified among the top 50 most cited articles.

Among the top 50 most cited articles, a total of 407 keywords were identified (Figure 4). The most used keyword was microbiology (*n* = 32), followed by endodontics (*n* = 26), peri-apical disease (*n* = 23), peri-apical periodontitis (*n* = 22), dentin (*n* = 18), tooth-pulp disease (*n* = 16), smear layer (*n* = 16), bacterial infection (*n* = 14), sodium hypochlorite (*n* = 13), anaerobic bacterium (*n* = 12), and root canal filling material (*n* = 9).

## 4. Discussion

The researchers and nonresearchers, like science journalists from various disciplines, are interested in knowing the milestone publications in their specialized field. This study was conducted to identify and characterise the top 50 most cited articles on the topic of endodontic microbiology published in endodontic journals. The oldest and the most recent articles were from the years 1965 and 2012, respectively. With international recognition and scientific outcomes in the specialized field, it is evident that both the article and the journal have contributed to the specialization [34]. A research article that has obtained the most frequent citation in its area of research is considered to have achieved a milestone in the field of scientific research [20]. Garfield states that an article which has secured 100 or more citations in the field of research could be considered as a classic article, depending on the speciality of research [35]. The articles included in the present study were cited more than 100 times. Hence, the top 50 articles are the classic articles in the field of endodontic microbiology.

The top 50 articles were cited between 103 and 1065 times and evaluated using the WoS all database as a benchmark. The WoS record suggests that less than 10% of its scientific articles remain uncited, probably even much less than that, because uncited articles in the database might have been cited by someone somewhere [36]. The WoS all database measures the scientific articles using an extensive period from 1945 to date. Contrastingly, Scopus and Google Scholar revealed fluctuations in the citation count. Scopus measures the citations starting from 1996, which is a severe flaw when evaluating the most cited articles, and Google Scholar covers all thesis, dissertation, reports, preprints, conference, and books which affects the scientific article counts in journals [37].

Publication year acts as an important factor with the citation count of an article; the citation of a scientific publication mostly follows a time-lapse. It is usually not cited until 1-2 years after the publication, reaching a peak in 3-10 years and then drops [38]. The older articles get ample time to be recognized, will be at a higher chance of getting cited when compared with the recently published ones [9, 13]. Hence, the recently published articles despite having a significant finding could not be identified. Furthermore, such studies achieve fewer citations, and the contribution has not been recognized universally. This could be the reason why the recently published articles have not been identified in the top 50 articles, and it is known as “obliteration by incorporation” effect [39]; hence, citation density of each article was calculated to overcome this bias. This study revealed that the first three most cited articles were published in the years 1965 to 1998, and most articles were from 2001, 2002, and 2003 contributing to 5 articles from each year. The differences in the publication rate in the years 2001 to 2003 may be due to the advancement in materials and techniques promoting research and scientific growth. One of the important feature of this study was the presence of two studies among the top 50 articles which were published in the last 10 years, i.e., 2012 and 2010 [32, 40]; this highlights the quality of the research and its relevance to the microbiology in endodontics with clinical implementation.

The findings of this study are in accordance with other bibliometric analyses performed within dentistry, suggesting that the institutes from the United States of America (USA) were involved in the top 50 most cited articles [3, 14, 37, 41–43]. The huge financial resources and the presence of a big scientific population and its active researcher community clarify the chief contribution of the USA [44]. It is important to mention that 24 articles are from European countries which are identified in the top 50 as international collaboration. In respect to research articles by the institution, Sweden, Switzerland, and Brazil with 5 articles, each was top on the list. Whereas research papers compared by individual authors, Siqueira Jr JF from Brazil was top on the list. A single article from Asian countries was identified and no contribution from African and Middle Eastern countries. Worldwide publication activity shows that the countries having low to middle income have a lower level of scientific articles published in high impact factor medical journals [45]. This could be due to difficulties in research and education, healthcare systems, limitations in achieving publications, lack of access to the information, and language barrier. Hence, further concern and research related to the microbiology in endodontics in the developing countries of the world is needed.

The journals' ranking based on their impact factor has become an important factor to consider when authors decide where to submit their research. The impact factor is corrupted as a proxy for the quality of individual publications [12]. Usually, authors target journals with the highest impact factor instead of journals having the best readers for their article [12]. Using bibliometric analysis to evaluate scientific evidence is a complex task; therefore, ranking journals using a single index would yield inaccurate results. Scientific publications must be evaluated keeping the impact factor aside. Impact factors can be misleading at times as general dentistry journals can have a much higher impact compared to their specialized counterparts. ES is becoming increasingly valuable as it focusses on the importance of specific papers, but there is dependence to a great extent on citation count which is a major limitation. Other bibliometric indexes are less predictable as they do not consider the quality of the evidence published [12].

The study design is linked to the contribution of the research, which marks the level of evidence. Depending on evidence-based practice, and the research design hierarchy suggests that the importance is given to the high graded studies like a systematic review, cohort studies, and randomized clinical trials [46]. In this analysis, the majority of the articles were on original research (both *in vivo* and *in vitro* studies) followed by narrated reviews and only one systematic review and meta-analysis [47]. The review articles in the list indicate that the researchers inclined towards gathering the existing research information and data in the field of microbiology in endodontics to provide benefits to the readers. The present study did not identify any randomized clinical trial (RCT). A study conducted by Crumley et al. (2005) revealed the most common reasons RCT or controlled clinical trial articles were missed in the electronic search were due to inadequate or inappropriate indexing. Additionally, why articles were not recognized in a database, includes they were published as reports, letters, books, book reviews, supplements, etc., or authors did not report keywords or methodology in the manuscript, and articles were missing from resources [48].

Keywords are an important component of a research article; on conducting a literature search, the use of keyword retrieves the more relevant results when compared with the use of sentences or phrases. The keywords act as code to source the required scientific articles [49]. The most used keywords were smear layer, root canal therapy, *Enterococcus faecalis*, endodontic re-treatment, endodontic failure, microbiology, and root canal infection. Several articles in this study did not contain the keywords. The literature suggests that the authors submitted the manuscript to the database with the keywords, but the published articles did not display [9]. The purpose of identifying the keywords is that it will guide and assist the researchers in searching for scientific papers in relevance to the microbiology in endodontics in different search engines.

## 5. Limitations

The limitation of this study was that the search was confined to the endodontic category of the WoS database. Studies on microbiology in endodontics can be published in nonendodontic journals focusing on microbiology and smear layer. Citation analysis is a fair technique for scientific article recognition. However, it does not consider the self-citation and negative citations [50]. This study includes only the top 50 articles due to the time limitation, which resulted in the exclusion of many articles from the list of classic articles. Hence, the top 50 articles which achieved the maximum citation were included in this study. The most recently published articles are at a disadvantage, regardless of the quality and the content of the paper as they were out of time criteria consideration. Future studies can be planned to use a broader category to include both endodontic and nonendodontic journals for inclusion of more articles.

## 6. Conclusion

The present study provided a detailed list of the top 50 most cited and classic articles on microbiology in endodontics. This will help researchers, students, and clinicians in the field of endodontics as an impressive source of information. The citation analysis provides the quantitative analysis of the scientific articles but not the quality or the important content of the article.

## Figures and Tables

**Table 1:** The top 50 published articles on endodontic microbiology.

Title of the article	Citation count (Web of Science)	Citation count (Elsevier Scopus)	Citation count (Google Scholar)	Current citation index (2019)	Citation density
(1) Kakehashi S, Stanley H, Fitzgerald R. 1965. “The Effects of Surgical Exposures of Dental Pulps in Germ-Free and Conventional Laboratory Rats.” Oral Surgery, Oral Medicine, Oral Pathology. 20 : 340-49.	1065	1202	2762	41	19.72
(2) Sundqvist G, Figdor D, Persson S, Sjögren U. 1998. “Microbiologic Analysis of Teeth with Failed Endodontic Treatment and the Outcome of Conservative Re-treatment.” Oral Surgery, Oral Medicine, Oral Pathology, Oral Radiology, and Endodontology. 85 : 86-93.	791	839	1883	26	37.67
(3) Molander A, Reit C, Dahlen G, Kvist T. 1998. “Microbiological Status of Root-Filled Teeth with Apical Periodontitis.” International Endodontic Journal. 31 : 1-7.	539	576	1269	14	25.67
(4) Nair PN, Henry S, Cano V, Vera J. “Microbial Status of Apical Root Canal System of Human Mandibular First Molars with Primary Apical Periodontitis after “One-Visit” Endodontic Treatment.” Oral Surgery, Oral Medicine, Oral Pathology, Oral Radiology, and Endodontology. 2005; 99 : 231-52.	437	432	920	26	31.21
(5) Stuart CH, Schwartz SA, Beeson TJ, Owatz CB. 2006. “Enterococcus faecalis: Its role in Root Canal Treatment Failure and Current Concepts in Retreatment.” Journal of Endodontics. 32 : 93-98.	432	480	1107	36	33.23
(6) Nair P (2006) “On the Causes of Persistent Apical Periodontitis: A Review.” International Endodontic Journal 39, 249–81.	383	379	901	23	29.46
(7) Nair PR, Sjögren U, Krey G, Kahnberg K-E, Sundqvist G. “Intraradicular Bacteria and Fungi in Root-Filled, Asymptomatic Human Teeth with Therapy-Resistant Periapical Lesions: A Longterm Light and Electron Microscopic Follow-Up Study.” J Endod 1990; 16 : 580-88.	377	414	925	7	13.00
(8) Siqueira Jr JF, Rôças IN. “Clinical Implications and Microbiology of Bacterial Persistence after Treatment Procedures.” J Endod 2008; 34 : 1291-1301.	321	385	770	44	29.18
(9) Pinheiro E, Gomes B, Ferraz C, Sousa E, Teixeira F, Souza-Filho F. 2003. “Microorganisms from Canals of Root-Filled Teeth with Periapical Lesions.” International Endodontic Journal. 36 : 1-11.	296	301	700	09	18.50
(10) Love R. 2001. “Enterococcus faecalis–A Mechanism for Its Role in Endodontic Failure.” International Endodontic Journal. 34 : 399-405.	282	312	767	24	15.67
(11) Rôças IN, Siqueira Jr JF, Santos KR. 2004. “Association of Enterococcus faecalis with Different Forms of Periradicular Diseases.” Journal of Endodontics. 30 : 315-20.	281	310	663	24	18.73
(12) Torabinejad M, Khademi AA, Babagoli J, Cho Y, Johnson WB, Bozhilov K, et al. 2003. “A New Solution for the Removal of the Smear Layer.” Journal of Endodontics. 29 : 170-75.	280	341	775	09	17.50
(13) Siqueira Jr JF, Rôças IN. 2004. “Polymerase Chain Reaction–Based Analysis of Microorganisms Associated with Failed Endodontic Treatment.” Oral Surgery, Oral Medicine, Oral Pathology, Oral Radiology, and Endodontology. 97 : 85-94.	270	288	586	12	18.00
(14) Hoshino E, Kurihara-Ando N, Sato I, Uematsu H, Sato M, Kota K et al. (1996) “In Vitro Antibacterial Susceptibility of Bacteria Taken from Infected Root Dentine to a Mixture of Ciprofloxacin, Metronidazole and Minocycline.” International Endodontic Journal 29, 125–30.	255	305	684	20	11.09
(15) Torabinejad M, Handysides R, Khademi AA, Bakland LK. 2002. “Clinical Implications of the Smear Layer in Endodontics: A Review.” Oral Surgery, Oral Medicine, Oral Pathology, Oral Radiology, and Endodontology. 94 : 658-66.	249	302	743	12	14.65
(16) Sundqvist G. 1992. “Ecology of the Root Canal Flora.” Journal of Endodontics. 18 : 427-30.	234	255	639	04	8.67
(17) Byström A, Happonen RP, Sjögren U, Sundqvist G. 1987. “Healing of Periapical Lesions of Pulpless Teeth after Endodontic Treatment with Controlled Asepsis.” Dental traumatology. 3 : 58-63.	226	259	535	06	7.06
(18) Hancock III H, Sigurdsson A, Trope M, Moiseiwitsch J. 2001. “Bacteria Isolated after Unsuccessful Endodontic Treatment in a North American Population.” Oral Surgery, Oral Medicine, Oral Pathology, Oral Radiology, and Endodontology. 91 : 579-86.	225	234	540	09	12.50
(19) Peciuliene V, Reynaud A, Balciuniene I, Haapasalo M (2001) “Isolation of Yeasts and Enteric Bacteria in Root-Filled Teeth with Chronic Apical Periodontitis.” International Endodontic Journal 34, 429–34.	218	221	566	08	12.11
(20) Sundqvist G. 1994. “Taxonomy, Ecology, and Pathogenicity of the Root Canal Flora.” Oral Surgery, Oral Medicine, Oral Pathology. 78 : 522-30.	208	218	547	06	8.32
(21) Sundqvist G, Johansson E, Sjögren U. 1989. “Prevalence of Black-Pigmented Bacteroides Species in Root Canal Infections.” Journal of Endodontics. 15 : 13-9.	203	205	432	01	6.77
(22) Distel JW, Hatton JF, Gillespie MJ. 2002. “Biofilm Formation in Medicated Root Canals.” Journal of Endodontics. 28 : 689-93.	198	200	468	08	11.65
(23) Nair PR. “Light and Electron Microscopic Studies of Root Canal Flora and Periapical Lesions.” Journal of Endodontics. 1987; 13 : 29-39.	182	207	504	08	5.69
(24) Violich D, Chandler N (2010) “The Smear Layer in Endodontics–A Review.” International Endodontic Journal 43, 2–15.	175	219	520	19	19.44
(25) Siqueira Jr JF. 2002. “Endodontic Infections: Concepts, Paradigms, and Perspectives.” Oral Surgery, Oral Medicine, Oral Pathology, Oral Radiology, and Endodontology. 94 : 281-93.	174	175	455	08	10.24
(26) Şen B, Wesselink P, Türkün M. 1995. “The Smear Layer: A Phenomenon in Root Canal Therapy.” International Endodontic Journal. 28 : 141-48.	168	168	505	02	7.00
(27) Baumgartner JC, Falkler WA. 1991. “Bacteria in the Apical 5 mm of Infected Root Canals.” Journal of Endodontics. 17 : 380-83.	166	175	443	03	5.93
(28) Peters LB, Wesselink PR, Buijs JF, Van Winkelhoff AJ. “Viable Bacteria in Root Dentinal Tubules of Teeth with Apical Periodontitis.” Journal of Endodontics. 2001; 27 : 76-81.	165	162	371	06	9.17
(29) Siqueira Jr JF. “Microbial Causes of Endodontic Flare-Ups.” International Endodontic Journal. 2003, 36 : 453-63.	160	194	485	17	10.00
(30) Peciuliene V, Balciuniene I, Eriksen HM, Haapasalo M. “Isolation of Enterococcus faecalis in Previously Root-Filled Canals in a Lithuanian Population.” Journal of Endodontics. 2000; 26 : 593-95.	157	171	385	05	8.26
(31) George S, Kishen A, Song P. “The Role of Environmental Changes on Monospecies Biofilm Formation on Root Canal Wall by Enterococcus faecalis.” Journal of Endodontics. 2005; 31 : 867-72.	154	181	324	13	11.00
(32) Siren EK, Haapasalo MP, Ranta K, Salmi P, Kerosuo EN. “Microbiological Findings and Clinical Treatment Procedures in Endodontic Cases Selected for Microbiological Investigation.” International Endodontic Journal. 1997; 30 : 91-95.	152	157	377	00	6.91
(33) Sen BH, Piskin B, Demirci T. “Observation of Bacteria and Fungi in Infected Root Canals and Dentinal Tubules by SEM.” Dental Traumatol. 1995; 11 : 6-9.	143	157	371	02	5.69
(34) Torabinejad M, Cho Y, Khademi AA, Bakland LK, Shabahang S. 2003. “The Effect of Various Concentrations of Sodium Hypochlorite on the Ability of MTAD to Remove the Smear Layer.” Journal of Endodontics. 29 : 233-39.	136	156	404	00	8.50
(35) Spratt D, Pratten J, Wilson M, Gulabivala K. 2001. “An In Vitro Evaluation of the Antimicrobial Efficacy of Irrigants on Biofilms of Root Canal Isolates.” International Endodontic Journal. 34 : 300-07.	125	140	344	02	6.94
(36) Siqueira Jr JF, Rôças IN. “Exploiting Molecular Methods to Explore Endodontic Infections: Part 1—Current Molecular Technologies for Microbiological Diagnosis.” Journal of Endodontics. 2005; 31 : 411-23.	123	121	202	11	8.79
(37) Shahravan A, Haghdoost A-A, Adl A, Rahimi H, Shadifar F. 2007. “Effect of Smear Layer on Sealing Ability of Canal Obturation: A Systematic Review and Meta-analysis.” Journal of Endodontics. 33 : 96-105.	122	147	361	10	10.17
(38) Takeda F, Harashima T, Kimura Y, Matsumoto K. 1999. “A Comparative Study of the Removal of Smear Layer by Three Endodontic Irrigants and Two Types of Laser.” International Endodontic Journal. 32 : 32-39.	121	140	356	02	6.05
(39) Kokkas AB, Boutsioukis AC, Vassiliadis LP, Stavrianos CK. 2004. “The Influence of the Smear Layer on Dentinal Tubule Penetration Depth by Three Different Root Canal Sealers: An In Vitro Study.” Journal of Endodontics. 30 : 100-02.	118	144	261	07	7.87
(40) Lottanti S, Gautschi H, Sener B, Zehnder M. 2009. “Effects of Ethylenediaminetetraacetic, Etidronic and Peracetic Acid Irrigation on Human Root Dentine and the Smear Layer.” International Endodontic Journal. 42 : 335-43.	117	129	237	18	11.70
(41) Teixeira C, Felippe M, Felippe W. 2005. “The Effect of Application Time of EDTA and NaOCl on Intracanal Smear Layer Removal: An SEM Analysis.” International Endodontic Journal. 38 : 285-90.	115	141	303	08	8.21
(42) Sunde PT, Olsen I, Debelian GJ, Tronstad L. 2002. “Microbiota of Periapical Lesions Refractory to Endodontic Therapy.” Journal of Endodontics. 28 : 304-10.	114	116	303	06	6.71
(43) Peters LB, Wesselink PR, Moorer WR. “The Fate and the Role of Bacteria Left in Root Dentinal Tubules.” International Endodontic Journal. 1995; 28 : 95-99.	114	131	278	04	4.75
(44) Chavez De Paz LE, Dahlén G, Molander A, Möller Å, Bergenholtz G. “Bacteria Recovered from Teeth with Apical Periodontitis after Antimicrobial Endodontic Treatment.” International Endodontic Journal. 2003; 36 : 500-08.	112	120	269	07	7.00
(45) Vera J, Siqueira Jr JF, Ricucci D, Loghin S, Fernández N, Flores B, Cruz AG. “One-Versus Two-Visit Endodontic Treatment of Teeth with Apical Periodontitis: A Histobacteriologic Study.” Journal of Endodontics. 2012; 38 : 1040-52.	112	121	271	13	16.00
(46) Noiri Y, Ehara A, Kawahara T, Takemura N, Ebisu S. “Participation of Bacterial Biofilms in Refractory and Chronic Periapical Periodontitis.” Journal of Endodontics. 2002; 28 : 679-83.	109	97	272	03	6.41
(47) Ando N, Hoshino E. “Predominant Obligate Anaerobes Invading the Deep Layers of Root Canal Dentine.” International Endodontic Journal. 1990; 23 : 20-27.	108	122	273	01	3.72
(48) Tronstad L, Barnett F, Cervone F. 1990. “Periapical Bacterial Plaque in Teeth Refractory to Endodontic Treatment.” Dental Traumatology. 6 : 73-77.	107	123	315	00	3.69
(49) Wittgow Jr WC, Sabiston Jr CB. 1975. “Microorganisms from Pulpal Chambers of Intact Teeth with Necrotic Pulps.” Journal of Endodontics. 1 : 168-71.	103	121	230	02	2.34
(50) O'Connell MS, Morgan LA, Beeler WJ, Baumgartner JC. 2000. “A Comparative Study of Smear Layer Removal Using Different Salts of EDTA.” Journal of Endodontics. 26 : 739-43.	103	109	270	06	5.42

**Table 2:** Journal impact factor, CiteScore, Eigenfactor, and other bibliometrics of the journals contributing to the top 50 most cited articles.

Journal name	Self-citations	Rank	Highest percentile (%)	Citations (2016-2019)	Documents (2016-2019)	5-year JIF^∗^	CiteScore	Eigenfactor score	No.of articles
J Endod	69	11/91	96	6884	1110	3.380	6.2	0.016	22
Int Endod J	50	6/91	97	3595	577	3.418	6.2	0.009	17
Oral Surg Oral Med	—	—	—	—	—	—	—	—	8
Oral Pathol Oral									
Radiol Endod									
Dent Traumatol	6	57/91	76	841	267	1.542	3.1	0.002	3

**Table 3:** Simple linear regression analysis of different journal metrics.

Variables	Coefficient standard error	Standardized coefficient beta	*p* value	95% CI Lower bound upper bound	
Self-citation	2.013	0.221	0.041^∗^	0.491	11.802
Highest percentile	22.133	0.085	0.714	-48.387	68.538
Documents	2.471	0.844	0.930	1.355	4.284
Cited percentage	33.098	0.143	0.475	-68.394	98.473
5-year JIF	317.364	-0.473	0.273	-1190.384	475.102
CiteScore	290.294	0.847	0.033^∗^	76.875	1578.283
Eigenfactor score	46877.294	0.475	0.006^∗^	36474.464	319806.112

## Data Availability

The data used to support the findings of this study are available from the corresponding author upon request.

## References

[1] Fouad A. F. (2017). Endodontic microbiology and pathobiology: current state of knowledge. *Dental Clinics*.

[2] Siqueira J., Rôças I. (2005). Uncultivated phylotypes and newly named species associated with primary and persistent endodontic infections. *Journal of Clinical Microbiology*.

[3] Fardi A., Kodonas K., Gogos C., Economides N. (2011). Top-cited articles in endodontic journals. *Journal of Endodontics*.

[4] Weiger R., De Lucena J., Decker H., Löst C. (2002). Vitality status of microorganisms in infected human root dentine. *International Endodontic Journal*.

[5] Neelakantan P. (2018). Endodontic microbiology—a special issue of dentistry journal. Multidisciplinary Digital Publishing Institute. *Dentistry Journal*.

[6] Prada I., Micó-Muñoz P., Giner-Lluesma T., Micó-Martínez P., Collado-Castellano N., Manzano-Saiz A. (2019). Influence of microbiology on endodontic failure. Literature review. *Medicina oral, patologia oral y cirugia bucal*.

[7] Farber P. A., Seltzer S. (1988). Endodontic microbiology. I. Etiology. *I. Etiology. Journal of endodontics*.

[8] de Paz L. E. C., Dahlén G. (2017). Microbiology and immunology of endodontic infections. *Endodontic Prognosis*.

[9] Arshad A. I., Ahmad P., Karobari M. I. (2020). Antibiotics: a bibliometric analysis of top 100 classics. *Antibiotics*.

[10] Moed H. F. (2002). The impact-factors debate: the ISI’s uses and limits. *Nature*.

[11] Garfield E. (2006). Citation indexes for science. A new dimension in documentation through association of ideas. *International journal of epidemiology*.

[12] Roldan-Valadez E., Orbe-Arteaga U., Rios C. (2018). Eigenfactor score and alternative bibliometrics surpass the impact factor in a 2-years ahead annual-citation calculation: a linear mixed design model analysis of radiology, nuclear medicine and medical imaging journals. *La Radiologia Medica*.

[13] Feijoo J. F., Limeres J., Fernández-Varela M., Ramos I., Diz P. (2014). The 100 most cited articles in dentistry. *Clinical Oral Investigations*.

[14] Gondivkar S. M., Sarode S. C., Gadbail A. R., Gondivkar R. S., Chole R., Sarode G. S. (2018). Bibliometric analysis of 100 most cited articles on oral submucous fibrosis. *Journal of Oral Pathology & Medicine*.

[15] Andersen J., Belmont J., Cho C. T. (2006). Journal impact factor in the era of expanding literature. *Journal of microbiology, immunology, and infection= Wei mian yu gan ran za zhi*.

[16] Shuaib W., Acevedo J. N., Khan M. S., Santiago L. J., Gaeta T. J. (2015). The top 100 cited articles published in emergency medicine journals. *The American Journal of Emergency Medicine*.

[17] Coats A. J. (2005). *Top of the charts: download versus citations in the International Journal of Cardiology*.

[18] Tam W. W., Wong E. L., Wong F. C., Hui D. S. (2013). Citation classics: top 50 cited articles in ‘respiratory system’. *Respirology*.

[19] Corbella S., Francetti L., Taschieri S., Weinstein R., Del Fabbro M. (2017). Analysis of the 100 most-cited articles in periodontology. *Journal of Investigative and Clinical Dentistry*.

[20] Tarazona B., Lucas-Dominguez R., Paredes-Gallardo V., Alonso-Arroyo A., Vidal-Infer A. (2018). The 100 most-cited articles in orthodontics: a bibliometric study. *The Angle Orthodontist*.

[21] Ahmad P., Dummer P., Chaudhry A., Rashid U., Saif S., Asif J. (2019). A bibliometric study of the top 100 most-cited randomized controlled trials, systematic reviews and meta-analyses published in endodontic journals. *International Endodontic Journal*.

[22] Ahmad P., Alam M., Jakubovics N., Schwendicke F., Asif J. (2019). 100 years of the Journal of Dental Research: a bibliometric analysis. *Journal of Dental Research*.

[23] Ahmad P., Arshad A. I., Della B. E., Khurshid Z., Stoddart M. (2020). Systemic manifestations of the periodontal disease: a bibliometric review. *Molecules*.

[24] Ahmad P., Della Bella E., Stoddart M. J. (2020). Applications of bone morphogenetic proteins in dentistry: A Bibliometric Analysis. *BioMed research international*.

[25] Roldan-Valadez E., Salazar-Ruiz S. Y., Ibarra-Contreras R., Rios C. (2019). Current concepts on bibliometrics: a brief review about impact factor, Eigenfactor score, CiteScore, SCImago journal rank, source-normalised impact per paper, H-index, and alternative metrics. *Irish Journal of Medical Science*.

[26] Villaseñor-Almaraz M., Islas-Serrano J., Murata C., Roldan-Valadez E. (2019). Impact factor correlations with Scimago journal rank, source normalized impact per paper, Eigenfactor score, and the CiteScore in radiology, nuclear medicine & medical imaging journals. *La Radiologia Medica*.

[27] Van Eck N. J., Waltman L. (2010). Software survey: VOSviewer, a computer program for bibliometric mapping. *scientometrics*.

[28] Kakehashi S., Stanley H., Fitzgerald R. (1965). The effects of surgical exposures of dental pulps in germ-free and conventional laboratory rats. *Oral surgery, oral medicine, oral pathology*.

[29] Sundqvist G., Figdor D., Persson S., Sjögren U. (1998). Microbiologic analysis of teeth with failed endodontic treatment and the outcome of conservative re-treatment. *Oral Surgery, Oral Medicine, Oral Pathology, Oral Radiology, and Endodontology*.

[30] Molander A., Reit C., Dahlen G., Kvist T. (1998). Microbiological status of root-filled teeth with apical periodontitis. *International Endodontic Journal*.

[31] Siqueira J. F., Rôças I. N. (2008). Clinical implications and microbiology of bacterial persistence after treatment procedures. *Journal of endodontics*.

[32] Vera J., Siqueira J. F., Ricucci D. (2012). One- versus two-visit endodontic treatment of teeth with apical periodontitis: a histobacteriologic study. *Journal of Endodontics*.

[33] Murad M. H., Asi N., Alsawas M., Alahdab F. (2016). New evidence pyramid. *BMJ Evidence-Based Medicine*.

[34] Ahmad P., Dummer P., Noorani T., Asif J. (2019). The top 50 most-cited articles published in the International Endodontic Journal. *International Endodontic Journal*.

[35] Martínez M. A., Herrera M., López-Gijón J., Herrera-Viedma E. (2014). H-Classics: characterizing the concept of citation classics through H-index. *Scientometrics*.

[36] Van Noorden R. (2017). The science That’s. *Nature*.

[37] Ahmad P., Elgamal H. A. M. (2020). Citation classics in the Journal of Endodontics and a comparative bibliometric analysis with the most downloaded articles in 2017 and 2018. *Journal of Endodontics*.

[38] Callaham M., Wears R. L., Weber E. (2002). Journal prestige, publication bias, and other characteristics associated with citation of published studies in peer-reviewed journals. *JAMA*.

[39] Gupta A., Kennedy B., Meriwether K. V., Francis S. L., Cardenas-Trowers O., Stewart J. R. (2020). Citation classics: the 100 most cited articles in Urogynecology. *International Urogynecology Journal*.

[40] Violich D., Chandler N. (2010). The smear layer in endodontics–a review. *International Endodontic Journal*.

[41] Tarazona B., Vidal-Infer A., Alonso-Arroyo A. (2017). Bibliometric analysis of the scientific production in implantology (2009–2013). *Clinical Oral Implants Research*.

[42] Hui J., Han Z., Geng G., Yan W., Shao P. (2013). The 100 top-cited articles in orthodontics from 1975 to 2011. *The Angle Orthodontist*.

[43] Jafarzadeh H., Sarraf S. A., Andersson L. (2015). The most-cited articles in dental, oral, and maxillofacial traumatology during 64 years. *Dental Traumatology*.

[44] Ahmad P., Vincent A. P., Khursheed A. M., Ahmed A. J. (2020). A bibliometric analysis of the top 50 most cited articles published in the Dental Traumatology. *Dental Traumatology*.

[45] Catalá-López F., Aleixandre-Benavent R., Caulley L. (2020). Global mapping of randomised trials related articles published in high-impact-factor medical journals: a cross-sectional analysis. *Trials*.

[46] Daly J., Willis K., Small R. (2007). A hierarchy of evidence for assessing qualitative health research. *Journal of Clinical Epidemiology*.

[47] Shahravan A., Haghdoost A.-A., Adl A., Rahimi H., Shadifar F. (2007). Effect of smear layer on sealing ability of canal obturation: a systematic review and meta-analysis. *Journal of Endodontics*.

[48] Crumley E. T., Wiebe N., Cramer K., Klassen T. P., Hartling L. (2005). Which resources should be used to identify RCT/CCTs for systematic reviews: a systematic review. *BMC Medical Research Methodology*.

[49] Natarajan K., Stein D., Jain S., Elhadad N. (2010). An analysis of clinical queries in an electronic health record search utility. *International Journal of Medical Informatics*.

[50] MacRoberts M. H., MacRoberts B. R. (1989). Problems of citation analysis: a critical review. *Journal of the American Society for Information Science*.

